# Vaccination Coverage Cluster Surveys in Middle Dreib – Akkar, Lebanon: Comparison of Vaccination Coverage in Children Aged 12-59 Months Pre- and Post-Vaccination Campaign

**DOI:** 10.1371/journal.pone.0168145

**Published:** 2016-12-19

**Authors:** Rodolfo Rossi, Ramia Assaad, Arianna Rebeschini, Randa Hamadeh

**Affiliations:** 1 International Committee of the Red Cross, Health Department, Beirut, Lebanon; 2 Republic of Lebanon, Ministry of Public Health, Primary Health Care services, Halba, Lebanon; 3 Republic of Lebanon, Ministry of Public Health, Social Health Service & Primary Health Care Department, Immunization and Essential Drugs Program, Beirut, Lebanon; Public Health England, UNITED KINGDOM

## Abstract

**Introduction:**

With the high proportion of refugee population throughout Lebanon and continuous population movement, it is sensible to believe that, in particular vulnerable areas, vaccination coverage may not be at an optimal level. Therefore, we assessed the vaccination coverage in children under 5 in a district of the Akkar governorate before and after a vaccination campaign. During the vaccination campaign, conducted in August 2015, 2,509 children were vaccinated.

**Materials and Methods:**

We conducted a pre- and post-vaccination campaign coverage surveys adapting the WHO EPI cluster survey to the Lebanese MoPH vaccination calendar. Percentages of coverage for each dose of each vaccine were calculated for both surveys. Factors associated with complete vaccination were explored.

**Results:**

Comparing the pre- with the post-campaign surveys, coverage for polio vaccine increased from 51.9% to 84.3%, for Pentavalent from 49.0% to 71.9%, for MMR from 36.2% to 61.0%, while the percentage of children with fully updated vaccination calendar increased from 32.9% to 53.8%. While Lebanese children were found to be better covered for some antigens compared to Syrians at the first survey, this difference disappeared at the post-campaign survey. Awareness and logistic obstacles were the primary reported causes of not complete vaccination in both surveys.

**Discussion:**

Vaccination campaigns remain a quick and effective approach to increase vaccination coverage in crisis-affected areas. However, campaigns cannot be considered as a replacement of routine vaccination services to maintain a good level of coverage.

## Introduction and Background

It is well understood that the rates of infant and childhood morbidity and mortality are significantly reduced through the use of an Expanded Program of Immunisation (EPI), one of the most efficient and effective public health interventions available. However, vaccination coverage in children varies greatly worldwide, both between and within countries, with low and middle-income countries having lower coverage.[1] The World Health Organisation (WHO) and the United Nations Children’s Fund (UNICEF) jointly estimated that measles vaccination, for example, in the African Region reached 74% in 2013, while the estimates for the same year were 78% for South-East Asia and Eastern Mediterranean Regions, 90% for the Americas, 95% for European and 97% for Western Pacific Regions.[2]. Vaccination coverage should ideally reach levels that allow herd immunity to work in order to keep the propagation of the disease to a level that is no longer a public health problem when eradication or elimination are not achievable.[3,4] It is also known that country’s reports on vaccination coverages are less reliable than survey reports.[5,6,7]

Due to the conflict in Syria, which is in its fifth year, the United Nations High Commissioner for Refugees (UNHCR) estimates that in Lebanon there are over 1 million refugees. Therefore, Lebanon is experiencing the highest concentration of refugees worldwide. The Akkar governorate, situated in the northern part of the country, is one of the regions with the highest number of Syrian refugees.[8]

In early 2015, the International Committee of the Red Cross (ICRC) conducted a formative needs assessment of the governorate of Akkar, and identified the Middle Drieb, an area of 50 square kilometres with 13 municipalities, as the area of highest vulnerability. Health service mapping confirmed that there is only one public health facility run by the Ministry of Social Affairs (MoSA) to serve a population of 54,000 people, thirty percent of whom are Syrian refugees.[9]

The Lebanese Ministry of Public Health (MoPH) have been supporting vaccination activities in this area: regular vaccine supply is provided for free to the MoSA health centre and to a private health centre; gas and solar fridges were also given to guarantee the storage of vaccines for these health centres. Moreover, polio mop-up campaigns were carried out during the past 3 years in collaboration with UNICEF and WHO, and sensitisation campaigns about vaccination were done for the public during the past months.

The municipality however raised concerns that childhood vaccination coverage was not fully achieved; this assumption was based on the fact that there was only one public health centre which provides free of charge vaccination for a large and vulnerable population. Moreover, the WHO, with the MoPH, reported a recent outbreak of mumps in the governorate; 70 percent of reported cases were Lebanese children.[10] The public infrastructure and services have been overwhelmed with the increase in population and the prolonged duration of the crisis.

Although the vaccination coverage for the whole country was estimated to be at around 90% [11] or above for both pentavalent and polio, with an uneven distribution of facilities able to conduct vaccination, it may be sensible to believe that local coverages may be different from the country estimates. For this reason, the MoPH district health office in Akkar requested the ICRC’s support to conduct a survey in the Middle Drieb area of Akkar. This survey was conducted on May 13^th^ 2015, in joint collaboration between the MoPH, the ICRC and the Lebanese Red Cross (LRC).

Following the results of the survey, a door-to-door vaccination campaign was conducted by the MoPH, with the support of the ICRC and LRC, in middle-Dreib during the first two weeks of August 2015. The vaccination targeted children up to 5 years of age, who received all the missing doses of vaccines according to the MoPH vaccination calendar.[12]

Since demographic information about Lebanon, such as age structure of the population, is very difficult to obtain or estimate (the last population census, for example, was done in 1932 [13]), it was necessary to repeat a vaccination coverage survey after the vaccination campaign to estimate the increase in vaccination coverage in children. Therefore, the Lebanese MoPH, supported by the ICRC and LRC, conducted a post-campaign survey on the 4^th^ November 2015.

The aim of this paper is to compare the increase of vaccination coverage in children 12–59 months following a vaccination campaign by reporting and comparing the results of first and second survey. The underlying hypothesis is that, when a door-to-door vaccination campaign is conducted (therefore trying to reach as many children as possible), the overall coverage for each vaccine may reach, as close as possible, the herd immunity threshold and limit the risk of outbreaks.

## Materials and Methods

### Ethical considerations

For both surveys, participating households were asked verbal consent to participate in the survey after they received a brief but thorough explanation of its aim. Verbal consent was asked (instead of a written one) in order to fully preserve the anonymity of participants, even from the interviewing teams; this approach was approved by the ethical board. Participants were aware that they had the right to refuse to answer the questions. If a household representative refused to respond, the interviewer moved to another household until the cluster was completed with 7 children identified and interviewed. No names were asked from the participants, therefore it is impossible to link in any way the answers to a particular respondent. The questionnaire answer sheets were kept in a locked drawer; the electronic dataset was protected by a password. Following the submission of a proposal, the Ministry of Public Health approved the conducting of the first survey on the 20^th^ March 2015 and the post-vaccination survey on the 27^th^ October 2015 (ref: 4355), and the vaccination campaign on the 20^th^ July 2015.

### Materials

#### Study design and study population

For both surveys, a WHO-standard EPI cluster survey [14] was carried out where 30 clusters were randomly selected with systematic sampling. The probability for a cluster to be selected was proportional to the population size of each village. Within each cluster, 7 children were randomly selected to obtain a total sample size of 210 children.

The study population and sample included children aged between 12–59 months living in the Middle Dreib district in the Akkar governorate. Children were included independently of their sex and national origin (see below the section on variables). Only one child per interviewed household was included.

#### Definition of outcome and other variables

A child was considered as “fully vaccinated” if he or she had received all the vaccination doses for his/her age according to the Lebanese Ministry of Public Health (MoPH) vaccination calendar (S1 File), which include pentavalent (DPT+HIB+HepB), MMR and Polio.[15] Only Syrian children receive BCG vaccine, since this antigen is not part of the Lebanese vaccination calendar while it is part of the Syrian calendar. However, in this study, BCG coverage was not analysed, nor considered to classify a child as fully vaccinated.

Other variables included sex of the child, age in months, nationality (resident or returnee Lebanese, Syrian, Palestinian or other), type of housing (house/apartment, tent settlement, collective shelter or other), whether the child had an immunization card or not. For each vaccination dose, we also recorded whether the dose was given in a hospital, a private health centre, a public primary health centre or by a mobile clinic or during a vaccination campaign. For children who did not have a complete vaccination calendar, the interviewers also asked about the reasons for not completing the vaccination. The possible reasons were:

- Lack of information concerning vaccination availability, MoPH vaccination calendar, need for boosters, etc.
- Personal beliefs about vaccination and fear of side effects
- Obstacles, such as cost, transport, unavailability of vaccine in the health centre, etc.
- Security
- Other

Vaccination status was primarily ascertained through the reading of the child’s vaccination card if available; alternatively, the mother’s recall was used to ascertain whether the child had received any given vaccine dose.

During the second survey, participants were also asked whether they moved to the Middle Dreib before or after the vaccination campaign (to assess whether population movement was associated with vaccination status) and whether they had heard/seen advertisement of the campaign that was conducted in August 2015.

#### Preliminary/preparatory steps: selection of clusters and other issues

The estimate population of each of the 13 municipalities of the Middle Dreib was obtained from the “Union of Municipalities” of Kouashra, one of the main villages of the Middle Dreib. The estimated figures obtained could give the proportion of Syrian refugees living in each village.

Each of the 13 villages were listed along their respective estimated population. Cumulative population was then calculated and divided by the number of cluster (30) to obtain the sampling interval. A random number was selected from a “random numbers table” to identify the first cluster. The identification of the subsequent clusters was obtained by adding each time the result for the sampling interval.

Since some villages had more than one cluster, satellite maps from Google were printed for each villages, and most central/known points (e.g. municipalities, mosques, market places, famous schools or monuments) were selected as starting point per each cluster. In each of these point, the survey team span a pen to randomly define the direction to find the 7 children.

The survey questionnaire was adapted by the WHO standard questionnaire for EPI survey.[14] The questionnaire was tested with 8 mothers exiting a Primary Care centre near the area of interest, amended, and then translated into Arabic and back into English.

40 interviewers (15 from the ICRC, 15 from the LRC and 10 from the MoPH), before both surveys, received one full day training on the conduction of the survey, vaccination calendar, informed consent, asking questions etc. These 40 interviewers were divided into 20 pairs; 10 pairs had to interview one cluster each, while the other 10 pairs had to interview two clusters each (total 30 clusters). 3 supervisors (2 ICRC, 1 MoPH) were available to answer by phone potential questions from the interviewers during the survey. Logistics (transport and security for the field teams) was organised by the ICRC and LRC.

### Methods

#### Data management and statistical analysis

A Microsoft Excel sheet was prepared to enter all the data. Responses were numerically coded (S1 Dataset). One ICRC staff entered all the data, and another staff independently cross-checked each entry. The dataset was transferred to STATA 11 software for analysis.

For each survey separately, all variables were explored through tabulations and cross-tabulation with correspondent p-value from the χ² test. Percentages were reported with their 95% Confidence Interval (95%CI).

Logistic regression was used to explore possible (crude and adjusted) association between the vaccination status of children and the following variables: nationality, age, type of shelter, sex of the child, village. For the second survey, population movement and advertisement of the vaccination campaign were added in the regression models. For the multivariable analysis, we used a forward stepwise approach, where variables were added in the model one by one, starting from those that appeared as most significant in the crude analysis. These associations were expressed as Odds Ratios (OR). Variables of particular interest were also kept in the model even if did not appear to be associated with the outcome. The resulting p-value from the Wald Test was used to assess the strength of association.

To compare the results of the two surveys, difference in proportion between the vaccination coverage of the two surveys was calculated and reported along with the 95%CI and tested with the χ² Test. We considered p<0.05 as statistically significant, while p<0.1 as marginally significant.

### The vaccination campaign (organisation and performance)

We chose 10 teams to conduct the vaccination campaign. Each team consisted of a MoPH nurse (to administer vaccine doses) and an LRC volunteer (for tally and clerical work).

Before the campaign, a training on routine immunizations, drop out protocol, vaccination procedures and course of action took place.

An awareness campaign was conducted beforehand with posters distributed to municipalities, mosques, primary health care centres, schools and other public places. A car with megaphone announced the campaign in the concerned villages 2 days prior to the start. Mukhtars, Head of municipalities and sheiks were all consulted in order to ensure a smooth event.

The campaign was carried out during the first two weeks of August 2015. A door-to-door approach was chosen in order to cover pockets of non-immunized children at home and in collective shelters. During the campaign, 2509 children up to 5 years old were vaccinated; they received 4300 vaccine doses as follow: 511 doses of MMR1, 510 of MMR2, 156 of Pentavalent 1, 50 of Pentavalent 2, 65 of Pentavalent 3, 43 Pentavalent 4, 2500 of Oral Polio Vaccine (OPV), 49 of Measles (for children too young to receive the MMR1), 413 of DPT (for children aged 4–5 years who had completed the Pentavalent cycle), and 3 of Hepatitis B (new-born children too young to receive the Pentavalent 1). Children without a vaccination card were provided with one. During the campaign, mothers of children who received the 1^st^ and 2^nd^ dose of Pentavalent and OPV gave their phone contact which was handed over to the El Bire Health Centre for follow-up of subsequent doses.

## Results

### Clusters attribution and response rate

In both surveys, the random systematic sampling to identify the 30 clusters attributed at least one cluster in each of the 13 villages of the surveyed area. El Bire, which is the largest town, received 9 clusters, while all the other villages received between 1–3 clusters.

To reach the desired sample size (210 children) during the first survey, the teams had to approach 220 household; 10 household representatives refused to respond. This gives a response rate of 95.5%. Similarly, during the second survey, 221 households were approached to reach the 210 sample size (response rate 95.0%). These, however, are the “minimal” estimated response rates, since we could not ascertain whether the few persons who refused to respond were or not eligible to participate in the survey (i.e. whether they had children aged 12–59 months).

### Demographic characteristics

We noticed very little and not significant changes in the demographic characteristics between the two surveys. In both surveys, boys were slightly more represented than girls, and Lebanese more than Syrians. During the second survey, children aged 24–59 were more represented than children aged 12–23 months compared to the first survey. Ownership of immunisation card increase of over 11% (p = 0.01) from the pre-campaign survey to the post-campaign one (Table 1).

**Table 1:** Percentage distribution of children aged 12–59 months by selected background characteristics

Variable	1^st^ survey		2^nd^ survey		Difference 1 vs 2 survey (95%CI of the difference)	P-value *
	N	% (95%CI)	N	% (95%CI)		
**Gender**						0.492
Male	112	53.3 (46.5; 60.1)	118	56.2 (49.4; 63.0)		
Female	98	46.7 (39.9; 53.5)	92	43.8 (37.0; 50.6)	-3.33% (-6.18; 12.84)	
**Nationality**						0.373
Lebanese	127	60.5 (53.8; 67.1)	118	56.2 (49.4; 63.0)		
Syrian	83	39.5 (32.9; 46.5)	92	43.8 (37.0; 50.6)	+4.29% (-5.14; 13.71)	
**Housing**						0.631
House/apartment	164	78.1 (71.9; 83.5)	168	80.0 (74.5; 85.5)		
ITS/CS/other	46	21.9 (16.5; 28.1)	42	20.0 (14.5; 25.5)	-1.90% (-5.88; 9.69)	
**Vaccination card**						<0.001
Yes	137	65.2 (58.4; 71.7)	161	76.7 (70.9; 82.4)	+11.43% (2.81; 20.04)	
No	73	34.8 (28.3; 41.6)	49	23.3 (17.6; 29.1)		
**Age (in months)**						0.031
12–23	83	39.5 (32.9; 46.5)	62	29.5 (23.3; 35.7)		
24–59	127	60.5 (53.8; 67.1)	148	70.5 (64.3; 76.7)	+10.00% (0.96; 19.04)	

This is the legend of Table 1

Frequencies are unweighted (N = 210), percentages and their 95%CI are weighted.

*P-value from χ^2^ Test for the difference in proportion between first and second survey

### Vaccination status of children

#### Coverage of each vaccine dose

In both surveys, coverage of each vaccine decreased with subsequent doses, indicating a dropout from the first dose of a vaccine towards the subsequent doses.

Coverage of each vaccine dose substantially increased at the second survey compared to the first one. The highest increases are seen for booster doses: polio 4 increases from a coverage of 48% at the first survey to a coverage of 82.7% at the second one (increase of 34.6%, p<0.001). Similarly Pentavalent 4 increases from the 44% seen at the first survey to 70.4% at the second (increase of 26.3%, p<0.001). Both MMR1 and MMR2 increase of more than 23% in the second survey compared to the first. While at the pre-campaign survey Lebanese had higher coverage than Syrian for most doses, at the post-campaign survey, Syrians appeared to have achieved higher coverage than Lebanese for most of vaccinations. However, apart from a few exceptions, these differences, both at the pre- and post-campaigns, were not significant. Overall, the increase of coverage for each dose appeared to be more important and significant for Syrians compared to Lebanese (Tables 2, 3 and 4).

**Table 2:** percentages of each vaccine dose coverage in general and separated by nationality at the first survey

Vaccine dose	Overall	Lebanese	Syrian	
	% (95%CI)	% (95%CI)	% (95%CI)	p-value*
**Polio 1 (N = 210)§**	91.9 (88.2; 95.6)	92.9 (88.5; 97.4)	90.4 (84.0; 96.7)	0.507
**Polio 2 (N = 210)§**	84.3 (79.3; 89.2)	85.8 (79.8; 91.9)	81.9 (73.6; 90.2)	0.445
**Polio 3 (N = 210)§**	71.9 (65.8; 78.0)	76.4 (69.0; 83.8)	65.1 (54.8; 75.3)	0.074
**Polio 4 (N = 168)¶**	48.2 (40.6; 55.8)	54.5 (44.7; 64.2)	38.8 (27.1; 50.5)	0.047
**Pentavalent 1 (N = 210)§**	88.1 (83.7; 92.5)	89.0 (83.6; 94.4)	86.7 (79.5; 94.0)	0.626
**Pentavalent 2 (N = 210)§**	81.0 (75.6; 86.3)	80.3 (73.4; 87.2)	81.9 (73.6; 90.2)	0.771
**Pentavalent 3 (N = 210)§**	70.5 (64.3; 76.7)	74.1 (66.4; 81.6)	65.1 (54.8; 75.3)	0.164
**Pentavalent 4 (N = 168)¶**	44.0 (36.5; 51.6)	47.5 (37.8; 57.3)	38.8 (27.1; 50.5)	0.265
**MMR1 (N = 210)§**	65.7 (59.2; 72.2)	66.9 (58.7; 75.1)	63.9 (53.5; 74.2)	0.646
**MMR2 (N = 168)¶**	31.5 (24.4; 38.6)	35.6 (26.3; 45.0)	25.4 (15.0; 35.8)	0.161

This is the legend of Table 2

*P-value from χ^2^ Test for difference in proportion between Lebanese and Syrians

§ Children of all ages (12–59 months)

¶ Children aged from 19 months and older who should have received polio 4, Pentavalent 4 and MMR 2

**Table 3:** percentages of each vaccine dose coverage in general and separated by nationality at the second survey

Vaccine dose	Overall	Lebanese	Syrian	
	% (95%CI)	% (95%CI)	% (95%CI)	p-value*
**Polio 1 (N = 210)§**	98.1 (96.2; 99.9)	97.5 (94.6; 99.9)	98.9 (96.8; 99.9)	0.444
**Polio 2 (N = 210)§**	95.2 (92.3; 98.1)	94.1 (91.0; 98.9)	95.6 (91.5; 99.8)	0.803
**Polio 3 (N = 210)§**	90.0 (85.9; 94.1)	88.1 (82.3; 94.0)	92.4 (87.0; 97.8)	0.308
**Polio 4 (N = 179)¶**	82.7 (77.1; 88.3)	80.0 (72.0; 88.0)	85.7 (78.2; 93.2)	0.313
**Pentavalent 1 (N = 210)§**	95.7 (93.0; 98.5)	94.1 (89.8; 98.3)	97.8 (94.8; 99.9)	0.182
**Pentavalent 2 (N = 210)§**	90.5 (86.5; 94.5)	86.4 (80.3; 92.6)	95.7 (91.5; 99.8)	0.024
**Pentavalent 3 (N = 210)§**	85.2 (80.4; 90.1)	82.2 (75.3; 89.1)	89.1 (82.8; 95.5)	0.160
**Pentavalent 4 (N = 179)¶**	70.4 (63.6; 77.1)	71.6 (62.5; 80.6)	69.0 (59.2; 78.9)	0.711
**MMR1 (N = 210)§**	89.0 (84.8; 93.3)	87.3 (81.3; 93.3)	91.3 (85.5; 97.1)	0.355
**MMR2 (N = 179)¶**	55.1 (47.7; 62.4)	51.1 (41.0; 61.2)	59.2 (49.0; 70.0)	0.257

This is the legend of Table 3

*P-value from χ^2^ Test for difference in proportion between Lebanese and Syrians

§ Children of all ages (12–59 months)

¶ Children aged from 19 months and older who should have received polio 4, Pentavalent 4 and MMR 2

**Table 4:** difference between first and second survey in percentages of each vaccine dose coverage in general, and separated by nationality

Vaccine dose	Difference 2^nd^ vs 1^st^ survey					
	Overall§	p-value*	Lebanese§	p-value*	Syrians§	p-value*
Polio1	+6.2 (2.1;10.3)	0.004	+4.5 (-0.7;9.8)	0.100	+8.6 (1.9;15.2)	0.010
Polio2	+11.0 (5.2;16.7)	<0.001	+9.1 (1.8;16.3)	0.017	+13.7 (4.5;23.0)	0.004
Polio3	+17.8 (10.4;25.1)	<0.001	+11.2 (1.8;20.5)	0.022	+27.3 (15.7;38.9)	<0.001
Polio4	+34.6 (25.2;43.9)	<0.001	+25.8 (13.2;38.3)	<0.001	+46.9 (33.0;60.8)	<0.001
Penta1	+7.6 (2.5;12.8)	0.004	+5.1 (-1.8;12.0)	0.155	+11.1 (3.2;19.0)	0.005
Penta2	+9.5 (2.9;16.2)	0.005	+6.1 (-3.1;15.4)	0.199	+13.7 (4.5;23.0)	0.004
Penta3	+14.8 (6.9;22.6)	<0.001	+8.2 (-2.1;18.5)	0.122	+24.1 (12.0;36.1)	<0.001
Penta4	+26.3 (16.3;36.4)	<0.001	+24.1 (10.7;37.4)	<0.001	+30.2 (14.9;45.5)	<0.001
MMR1	+23.3 (15.6;31.0)	<0.001	+20.4 (10.2;30.5)	<0.001	+27.4 (15.6;39.3)	<0.001
MMR2	+23.5 (13.4;33.6)	<0.001	+15.4 (1.7;29.2)	0.030	+34.2 (19.4;48.9)	<0.001

This is the legend of Table 4

§Difference in proportion of vaccinated children per each antigen dose between the first and second survey

*P-value from the χ^2^ test for difference in proportions

#### Children with updated vaccination calendar per each antigen and overall

At the pre-campaign survey (Table 5), out of the 210 children, 69 (32.9%) were fully vaccinated, meaning that they received all the vaccine doses, for each antigen, foreseen by the MoPH vaccination calendar according to the children’s age.

**Table 5:** Percentage of fully vaccinated children aged 12–59 months for each vaccine and with a complete vaccination calendar, by selected background characteristics and overall at the pre-campaign survey

**Vaccinated for:**	**Polio**		**Pentavalent¶**		**MMR¥**		**Fully vaccinated§**	
**Characteristics**	% (95%CI)	p-value*	% (95%CI)	p-value*	% (95%CI)	p-value*	% (95%CI)	p-value*
**Gender:**		0.057		0.093		0.673		0.346
Male	58.0 (48.9; 67.2)		54.5 (45.2; 63.7)		37.5 (28.5; 46.5)		35.7 (26.8; 44.6)	
Female	44.9 (35.1; 54.7)		42.9 (33.1; 52.7)		34.7 (25.3; 44.1)		29.6 (20.6; 38.6)	
**Nationality:**		0.045		0.184		0.372		0.199
Lebanese	57.5 (48.9; 66.1)		52.8 (44.1; 61.4)		38.6 (30.1; 47.0)		36.2 (27.9; 44.6)	
Syrian	43.4 (32.7; 54.0)		43.4 (32.7; 54.0)		32.5 (22.5; 42.6)		27.7 (18.1; 37.3)	
**Child lives in:**		0.050		0.128		0.567		0.144
House/apartment	55.5 (47.9; 63.1)		51.8 (44.2; 59.5)		37.2 (29.8; 44.6)		35.4 (28.0; 42.7)	
ITS/CS/other	39.1 (25.0; 53.2)		39.1 (25.0; 53.2)		32.6 (19.1; 46.2)		23.9 (11.6; 36.2)	
**Village:**		0.320		0.217		0.004		0.008
El Bire#	57.1 (44.9; 69.4)		55.6 (43.3; 67.8)		50.8 (38.4; 63.1)		46.0 (33.7; 58.3)	
Elsewhere	49.7 (41.6; 57.7)		46.3 (38.2; 54.3)		29.9 (22.5; 37.3)		27.2 (20.0; 34.4)	
**Immunisation card:**		0.156		0.024		0.102		0.357
Yes	55.5 (47.2; 63.8)		54.7 (46.4; 63.1)		40.1 (31.9; 48.4)		35.0 (27.0; 43.0)	
No	45.2 (33.8; 56.6)		38.4 (27.2; 49.5)		28.8 (18.4; 39.2)		28.8 (18.4; 39.2)	
**Age (months):**		0.557		0.935		0.564		0.827
12–23	49.4 (38.6; 60.2)		49.4 (38.6; 60.2)		38.6 (28.1; 49.0)		33.7 (23.6; 43.9)	
24–59	53.5 (44.9; 62.2)		48.8 (40.1; 57.5)		34.6 (26.4; 42.9)		32.3 (24.2; 40.4)	
**Total/Overall**	51.9 (44.9; 58.8)	—	49.0 (42.1; 56.0)	—	36.2 (29.7; 43.1)	—	32.9 (26.5; 39.7)	—

This is the legend of Table 5

*P-value from the χ^2^ Test for difference in percentage

#El Bire is the only village in all Middle Dreib with a PHC public service

§To be fully vaccinated, a child needs to have received full course of Polio, Pentavalent and MMR according to his/her age.

¶Diphtheria, Pertussis, Tetanus, Haemo-Influenza B and Hepatitis B

¥Measles, Mumps, Rubella

ITS: Informal Tent Settlement

CS: Collective Shelter

The coverage of each vaccine varied; 51.9% (n = 109) of children were fully protected from polio; similarly, 49.05% (n = 103) received a full course of pentavalent, while 36.2% (n = 76) received the full course of MMR (i.e. one dose if the child is less than 18 months, two doses if the child is 18 months or older). In general, vaccination coverage was higher for Lebanese (compared to Syrians), for those living in a house/apartment (compared to those living in other informal settlements) and for boys (compared to girls), although these differences were not or only marginally significant. The only exception were children living in El Bire town versus other villages: in El Bire, children were generally better vaccinated in comparison to other villages (Table 5).

At the post-campaign survey (Table 6), 113 children (53.8%) were fully vaccinated. The coverage of each vaccine varied; 84.3% (n = 177) of children were fully protected from polio; 71.9% (n = 151) received a full course of pentavalent, while 60.9% (n = 128) received the full course of MMR (i.e. one dose if the child is less than 18 months, two doses if the child is 18 months or older). In line with the first survey, vaccination coverage was slightly higher for Lebanese (compared to Syrians) and for boys (compared to girls), although these differences were not significant. However, children living in collective shelter or tents were less vaccinated than children living in houses or apartments (p = 0.02). Also at the second survey, children living in El Bire town versus other villages were generally better vaccinated in comparison to other villages (Table 6).

**Table 6:** Percentage of fully vaccinated children aged 12–59 months for each vaccine and with a complete vaccination calendar, by selected background characteristics and overall at the post-campaign survey

**Vaccinated for:**	**Polio**		**Pentavalent¶**		**MMR¥**		**Fully vaccinated§**	
**Characteristics**	% (95%CI)	p-value*	% (95%CI)	p-value*	% (95%CI)	p-value*	% (95%CI)	p-value*
**Gender:**		0.331		0.505		0.380		0.888
Male	86.4 (80.3; 92.6)		73.7 (65.8; 81.7)		63.6 (54.8; 72.2)		54.2 (45.2; 63.2)	
Female	81.5 (73.6; 89.5)		69.6 (60.2; 79.0)		57.6 (47.5; 67.7)		53.3 (43.1; 63.5)	
**Nationality:**		0.578		0.505		0.792		0.675
Lebanese	83.1 (76.3; 89.8)		73.7 (65.8; 81.7)		60.2 (51.3; 69.0)		55.1 (46.1; 64.1)	
Syrian	85.7 (78.8; 93.0)		69.6 (60.2; 79.0)		62.0 (52.0; 71.9)		52.2 (42.2; 62.4)	
**Child lives in:**		0.107		0.002		0.203		0.022
House/apartment	86.3 (81.1; 91.6)		76.8 (70.4; 83.2)		63.1 (55.8; 70.4)		57.7 (50.3; 65.2)	
ITS/CS/other	76.2 (63.3; 89.1)		52.4 (37.3; 67.5)		52.4 (37.3; 67.5)		38.1 (23.4; 52.8)	
**Village:**		0.043		0.056		0.019		0.032
El Bire#	92.1 (85.4; 98.7)		81.0 (71.3; 90.6)		73.0 (62.1; 84.0)		60.1 (53.3; 76.9)	
Elsewhere	81.0 (74.6; 87.3)		68.0 (60.5; 75.6)		55.8 (47.8; 63.8)		49.0 (40.9; 57.1)	
**Immunisation card:**		0.560		0.124		0.338		0.904
Yes	85.1 (79.6; 90.6)		74.5 (67.8; 81.3)		62.7 (55.3; 70.2)		54.0 (46.3; 61.7)	
No	81.6 (70.8; 92.5)		63.3 (49.8; 76.8)		55.1 (41.2; 69.0)		53.1 (39.1; 67.0)	
**Age (months):**		0.915		0.137		0.025		0.020
12–23	83.9 (74.7; 93.0)		79.0 (68.9; 89.2)		72.6 (61.5; 83.7)		66.1 (54.3; 77.9)	
24–59	84.5 (78.6; 90.3)		68.9 (61.5; 76.4)		56.1 (48.1; 64.1)		48.6 (40.6; 56.7)	
**Total/Overall**	84.3 (79.3; 89.2)	—	71.9 (65.8; 78.0)	—	61.0 (54.3; 67.6)	—	53.8 (47.0; 60.6)	—

This is the legend of Table 6

*P-value from the χ^2^ Test for difference in percentage

#El Bire is the only village in all Middle Dreib with a PHC public service

§To be fully vaccinated, a child needs to have received full course of Polio, Pentavalent and MMR according to his/her age.

¶Diphtheria, Pertussis, Tetanus, Haemo-Influenza B and Hepatitis B

¥Measles, Mumps, Rubella

ITS: Informal Tent Settlement

CS: Collective Shelter

By comparing the results of the two surveys (Table 7), full coverage for each antigen increased across all the various categories. Overall, full coverage for Polio increased by 32.4% (p<0.001), Pentavalent by 22.9% (p<0.001), MMR by 24.8% (p<0.001) and the percentage of children with a completely updated vaccination calendar increased by 21.0% (p<0.001). By looking across the various categories, increase of full coverage for each antigen was less evident for children living in tent settlements and collective shelters. For these children, with the exception of polio which increased by 37.1% (p = 0.0005), the coverage for the other antigens was either marginally or not significant: coverage for Pentavalent increased by 13.3% (p = 0.21), MMR by 19.8 (p = 0.06) and the percentage of children with a completely updated vaccination calendar increased by 14.2% (p = 0.15).

**Table 7:** Change between pre- and post-campaign in percentage of fully vaccinated children aged 12–59 months for each vaccine and with a complete vaccination calendar, by selected background characteristics and overall

	Polio		Pentavalent		MMR		Fully vaccinated	
Characteristic	Diff% (95%CI)	p-value*	Diff% (95%CI)	p-value*	Diff% (95%CI)	p-value*	Diff% (95%CI)	p-value*
**Gender**								
Male	+28.5 (17.5;39.5)	<0.001	+19.5 (7.4;31.6)	0.002	+26.4 (13.9;38.8)	<0.001	+18.9 (6.3;31.5)	0.004
Female	+36.4 (23.7;49.1)	<0.001	+26.4 (12.7;40.0)	<0.001	+22.4 (8.6; 36.3)	0.002	+23.2 (9.5;36.8)	0.001
**Nationality**								
Lebanese	+25.6 (14.6;36.5)	<0.001	+21.0 (9.2;32.7)	<0.001	+21.6 (9.4;33.8)	<0.001	+18.9 (6.6;31.1)	0.003
Syrian	+42.5 (29.7;55.3)	<0.001	+26.2 (12.0;40.4)	<0.001	+29.4 (15.3;43.4)	<0.001	+24.5 (10.4;38.5)	0.001
**Accommodation**								
House/apart.	+30.8 (21.6;40.0)	<0.001	+25.0 (15.0;34.9)	<0.001	+25.9 (15.5;36.3)	<0.001	+22.4 (11.9;32.8)	<0.001
ITS/CS/Other	+37.1 (18.0;56.2)	<0.001	+13.3 (-7.4;33.9)	0.212	+19.8 (-0.5;40.1)	0.060	+14.2 (-5.0;33.4)	0.150
**Age**								
12–23	+34.5 (20.3;48.6)	<0.001	+29.6 (14.9;44.4)	<0.001	+34.0 (18.8;49.3)	<0.001	+32.4 (16.8;48.0)	<0.001
24–59	+30.9 (20.5;41.4)	<0.001	+20.1 (8.6;31.6)	<0.001	+21.4 (9.9;32.9)	<0.001	+16.4 (4.9;27.8)	0.006
**Tot/overall**	+32.4 (24.0;40.7)	<0.001	+22.9 (13.8;31.9)	<0.001	+24.8 (15.5;34.0)	<0.001	+21.0 (11.7;30.2)	<0.001

This is the legend of Table 7

*P-value from the χ^2^ Test for difference in percentage

#### Factors associated with complete vaccination calendar (result of logistic regression)

At the first survey, the logistic regression analysis, to assess which factors were associated with a child being fully vaccinated, revealed that the only strong factor was whether the child was living in El Bire compared to any other village (crude OR = 2.28; 95%CI 1.23 to 4.22; p = 0.008; and adjusted for nationality and type of shelter, OR = 2.55; 95%CI 1.35 to 4.82; p = 0.004). In the second survey, however, other factors were identified: while living in El Bire village remained strongly associated with having a completely updated vaccination calendar, younger children (aged 12–23 months) and children living in houses and/or apartments were also more likely to have their vaccination calendar completely updated (Table 8). Population movement and advertisement of the campaign, as well as nationality and child sex, did not seem to be statistically associated with the vaccination status of children (Table 8).

**Table 8:** factors associated with being fully vaccinated at the second survey. Measure expressed as Odds Ratio (OR)

Variable	Crude OR (95% CI)	p-value*	Adjusted OR (95% CI)#	p-value*
**Living Village:**				
El Bire	1.94 (1.05; 3.58)	0.033	2.19 (1.03; 4.63)	0.041
Elsewhere	Ref		Ref	
**Child lives in:**				
House/apartment	2.22 (1.11; 4.44)	0.024	3.17 (1.19; 8.44)	0.021
Tent/C. Shelter/other	Ref		Ref	
**Age (months):**				
12–23	2.06 (1.11; 3.82)	0.022	2.57 (1.22; 5.38)	0.013
24–59	Ref		Ref	
**Sex**				
Boys	1.04 (0.60; 1.80)	0.888	1.16 (0.61; 2.23)	0.650
Girls	Ref		Ref	
**Nationality**				
Lebanese	Ref		Ref	
Syrian	0.89 (0.51; 1.54)	0.675	1.15 (0.54; 2.47)	0.718
**Heard of campaign advertisement**				
No	Ref		Ref	
Yes	0.92 (0.41; 2.06)	0.835	1.61 (0.65; 4.00)	0.305
**Living in M.D. before the campaign**				
No	1.59 (0.52; 4.85)	0.413	2.02 (0.59; 6.92)	0.263
Yes	Ref		Ref	

This is the legend of Table 8

*P-value from Wald Test

#Adjusted for the other variables in the table

M.D.: Middle Dreib

### Reported sources/health facilities for vaccination

At the first survey, out of the 1,409 given vaccine doses that we recorded, 40.5% were provided by public Primary Health Care services, followed by the private health sector (32.7%), mobile clinics/campaigns (13.7%), and finally, secondary level of care (13.1%, virtually all and only the “0” dose of hepatitis B). At the second survey, of the 1,926 given vaccine doses that we recorded, 44.5% were provided by public Primary Health Care services, followed by the private health sector (18.8%), mobile clinics/campaigns (18.6%), and finally, secondary level of care (8.2%, virtually all and only the “0” dose of hepatitis B).

### Reported reasons for non/incomplete vaccination

In both surveys, the respondents reported lack of information (knowledge about boosters, necessity to immunize, where to get vaccines) as the primary reason for not completing the vaccination (39.1% and 38.3% at the first and second surveys, respectively)–Table 9.

**Table 9:** Reported reasons for incomplete vaccination calendar (N = 115 at the 1^st^ survey, N = 81 at the 2^nd^ survey)

Reported reason	1^st^ survey–n (%)	2^nd^ survey–n (%)
Information*	45 (39.1%)	31 (38.3%)
Personal belief§	13 (11.3%)	17 (21.0%)
Obstacles#	33 (28.7%)	28 (34.6%)
Security	1 (0.9%)	0 (0.0%)
Other	23 (20.0%)	5 (6.2%)

This is the legend of Table 9

* Information includes: awareness of need for immunisation and subsequent doses; awareness of where to get vaccinated

§ Personal belief also includes fear of side effects

#Obstacles includes: cost of service/transport, availability of vaccine, child too sick when at the clinic

## Discussion

### Summary of results

At the survey we performed in May (first survey), the vaccination coverage in the assessed area was found to be below a sufficient target. If protection is assumed with the completion of the due vaccination calendar (although fewer doses may still provide some immunity), for none of the vaccine-preventable diseases, the vaccination coverage was sufficient to reach a level for the herd immunity to work efficiently, leaving an important risk of outbreaks. The percentage of immune individuals to prevent an outbreak varies depending on the Basic Reproductive Number (R0), but it can be as high as 94% for measles and pertussis.[4] Coverage for polio was found slightly higher than for the other diseases probably related to the recent mop-up campaigns that were carried out by the MoPH during the previous months or weeks. The poorest level of coverage was for the Measles-Mumps-Rubella (MMR), for which just more of a third of the surveyed children were covered.

These results were not in line with the national estimated vaccination coverage.[11] We can also see that between the first and other doses (e.g. Polio 1 and 3) there was significant drop out, sign that children attended for the first dose only. We noticed that Lebanese nationals had a slight better coverage compared to Syrian refugees, although these differences were either marginally or not significant. Similarly, differences in coverage for persons living in houses/apartments compared to persons living in informal settlements (tents or collective shelters)–who are mostly Syrians–were not significant.

At the second survey, done 2 months after the vaccination campaign, however, we saw a great improvement in the general vaccination coverage and for each dose of all antigens. Although the coverage for MMR is still suboptimal (if completeness is considered with 2 doses), for most of the vaccine-preventable diseases, the coverage is very close to or within the herd immunity threshold. Coverage for polio is still found slightly higher than for the other diseases, likely to be related to the recent campaign in which all children receive a dose of OPV regardless their vaccination status. The poorest level of coverage was for the Measles-Mumps-Rubella (MMR), mostly for the second dose (MMR2). This lower coverage for MMR 2 is likely due to the recent changes of the MoPH vaccination calendar, in which the second dose of MMR is anticipated from the age of 4–5 years to 18 months. In fact, it is seen that children aged 12–23 had better coverage of MMR compared to children aged 24–59 months; this is likely due to the fact that these children received the first dose of MMR after the MoPH calendar was changed, therefore they were informed about the timing of the second dose.

No difference was noted in coverage between Lebanese and Syrians, although, in this second survey, Syrians appears to have improved their coverage to a larger extent compared to Lebanese children. However, children living in tent settlements or collective shelter still had a significant lower coverages of all vaccines compared to children living in houses or apartments.

In both surveys, children living in El Bire town (the only known public PHC service providing vaccination for free) were found to be more protected than children living in all the other villages. This may imply difficulties in access to reach the health centre and possible low demand of vaccination services from the population. These hypotheses are in line with the reported reasons for non/incomplete vaccination of these surveys. Population movement and advertisement of the campaign did not seem to be associated with the vaccination status of children.

To our knowledge, this has been the first survey to assess the impact on vaccination coverage, following a campaign, in Lebanon since the beginning of the Syrian crisis; therefore, we cannot compare the results of this study with others in the same context. However, vaccination campaigns have been proven successful for quickly increase the vaccination coverage in various context, such as Bangladesh [16] and India [17]. Although our study fails to show it, vaccination campaigns also appear have a positive promotional impact on the use of routine vaccination services.[18]

### Strengths and limitation of this study

The major strength of this study is that it followed a highly recommended and validated survey approach (the WHO standard EPI cluster survey).[14] Since we scrupulously followed the steps of this method, the risk of error was minimised. Another major strength is the very high response rate, which also minimised the risk of selection bias. The good reputation of the LRC and MoPH in the area and the fact that many LRC/MoPH volunteers were interviewers helped achieve such response rate. The comprehensive training done for the interviewers’ left very little margin of error concerning information bias, misclassification and missing values.

The major limitation of the study was that about one third (at the first survey, but only a quarter at the second one) of the children did not have an immunisation card and therefore the vaccination status had to be assessed through the mothers’ recall. The mothers could recall where the vaccine were given, therefore adding more credibility to their answers. However, “socially-acceptable” answers (resulting in possible information bias) from those without a card cannot be excluded, and the potential direction of misclassification (if any) cannot be foreseen. Another limitation of this study is that it failed in capturing whether the promotional activities done during the vaccination campaign had an effect in increased uptake of vaccination through the routine system at the health centres; although this is an important point, the answer should probably be sought through another study design. Similarly, the data of this study fail in allowing a deeper analysis on the facilities which provided the vaccination; since this point can have important public health implications, a different study design should be done to answer this question.

### Public health implications and recommendations

Our results are in line with other studies [19] showing that vaccination campaigns remain a quick and effective method to catch-up the vaccination status of children when this appears to be suboptimal. Vaccination campaigns may be a good approach to overcome some of the obstacles to have children vaccinated (as reported by respondents in both surveys). Once a campaign is organised, it should be mandatory that the full vaccination calendar is provided to targeted children; this is to optimise the resources allocated to vaccination programmes. Nevertheless, campaigns are logistically and financially challenging and cannot be considered as a replacement of vaccination routine services, which need to be reinforced if a good coverage is to be maintained. Free-of-charge vaccination should be promoted also in private primary health care centres in order to increase the opportunity for and access to vaccination, as also seen in other studies.[19,20]

In a country such as Lebanon, where there is very little demographic information and update (the last population census was done in 1932), it is important to perform a pre- and post-campaign surveys to assess the success of the vaccination campaign. The post-campaign coverage survey should be conducted within the best delay from the campaign.

Effectiveness of campaign advertisement should be assessed with more rigorous study designs in order to see whether advertisement is worth investing prior vaccination campaigns.

A high proportion of fully vaccinated children is very difficult to obtain in a situation of crisis, particularly if there are frequent population movements and an imbalance between the availability of primary care services and the population size; in spite of the emergency situation Lebanon is witnessing, the Lebanese MoPH, together with its partners succeeded to maintain a "polio free status" in the country. We see and appreciate the successful efforts of the MoPH to provide services to both Lebanese and Syrians.

## Supporting Information

S1 Dataset Anonymised Excel dataset. (ZIP)

S1 File Lebanese MoPH vaccination calendar. (PDF)
