# Supplementary material for: Vaccination Coverage Cluster Surveys in Middle Dreib – Akkar, Lebanon: Comparison of Vaccination Coverage in Children Aged 12-59 Months Pre- and Post-Vaccination Campaign
Source: PLoS One. 2016 Dec 19;11(12):e0168145. doi: 10.1371/journal.pone.0168145 (PMC5167265; doi:10.1371/journal.pone.0168145)
Supplement: S1 File — (PDF) [file pone.0168145.s002.pdf]

الجمهورية اللبنانية
وزارة الصحة العامة

National Immunization Schedule

Children 0-5 years of Age

Child Age	Vaccine	Dose
At Birth *(First **8 hours** of birth)*	Hep B	At the Hospital (0 Dose)
2 months	IPV (Polio), DPT- HIB- Hep B	1st Dose
4 months	OPV (Polio), DPT- HIB- Hep B	2nd Dose
6 months	OPV (Polio), DPT- HIB- Hep B	3rd Dose
9 months	Measles	“0” Dose
12 months	MMR	1st Dose
18 months	OPV Polio, DPT- HIB- Hep B	Booster Dose
	MMR	2nd Dose
4- 5 years	OPV Polio, DPT	Booster Dose

IPV: Inactivated polio vaccine
OPV: Oral Polio Vaccine
DPT: Diphtheria- Pertussis- Tetanus
Hep B: Hepatitis B
HIB: Haemophilus influenza type B
MMR: Measles- Mumps- Rubella
